# Multipolar adaptive traction allows diagnostic endoscopic submucosal dissection for colonic lesions with focal invasive area

**DOI:** 10.1055/a-2208-5432

**Published:** 2023-12-05

**Authors:** Pierre Lafeuille, Louis Jean Masgnaux, Timothée Wallenhorst, Jérémie Jacques, Alexandru Lupu, Jérôme Rivory, Mathieu Pioche

**Affiliations:** 1 36609 Gastroenterology, Groupement Hospitalier Edouard Herriot, Lyon, France; 2 36609 Endoscopy, Groupement Hospitalier Edouard Herriot, Lyon, France; 3 36684 Department of Endoscopy and Gastroenterology, University Hospital Centre Rennes, Rennes, France; 4 service d'hépato-gastro-entérologie, CHU Dupuytren Limoges, Limoges, France; 5 gastroenterology and endoscopy, Pavillon L Edouard Herriot Hospital, Lyon, France; 6 Gastroenterology, Edouard Herriot Hospital, Lyon, France

Endoscopic submucosal dissection (ESD) has become the gold standard for superficial colorectal neoplasia resection. As recently reported, diagnostic ESD is also feasible and safe for colorectal lesions with a focal deep invasive pattern of less than 15 mm 1 . But the R0 resection rate for these T1 cancers could be improved on the vertical margin. Although endoscopic intermuscular dissection has recently been described as a method of achieving free vertical margins by dissecting more deeply in the rectum 2 , this approach is not feasible in the colon where the two muscular layers are thin. To facilitate exposure of the submucosal space, which is often very thin when it remains, an adaptive traction device that is capable of being tightened to increase traction during the late stages of the procedure (A-TRACT-2; Hospices Civils de Lyon, France) could be useful 3 4 .

We report here the case of a 73-year-old patient with a 12-mm pseudodepressed non-granular laterally spreading tumor (LST) in the left colon, with a 6-mm Kudo Vn, Sano 3b demarcated area highly suspicious of deep invasion ( Fig. 1 ). After circumferential incision, the device was fixed with clips on the oral and anal edges. The device was then fixed to the opposite wall. When traction began to decline, the device was tightened, allowing better exposure on the very thin submucosal space, leading to an R0 resection with no adverse events ( Fig. 2 , Video 1 ).

**Fig. 1:**
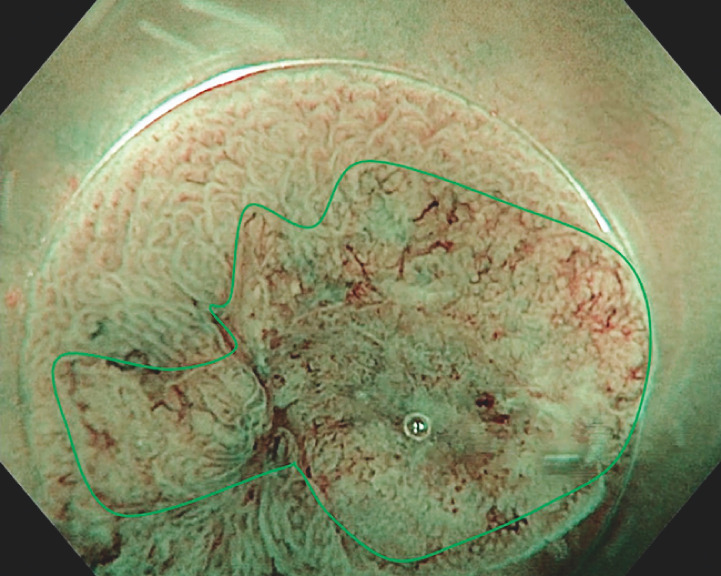
Endoscopic view of the non-granular laterally spreading tumor with a demarcated area highly suspicious of deep degeneration (bounded by the green line) on chromoendoscopy.

**Fig. 2:**
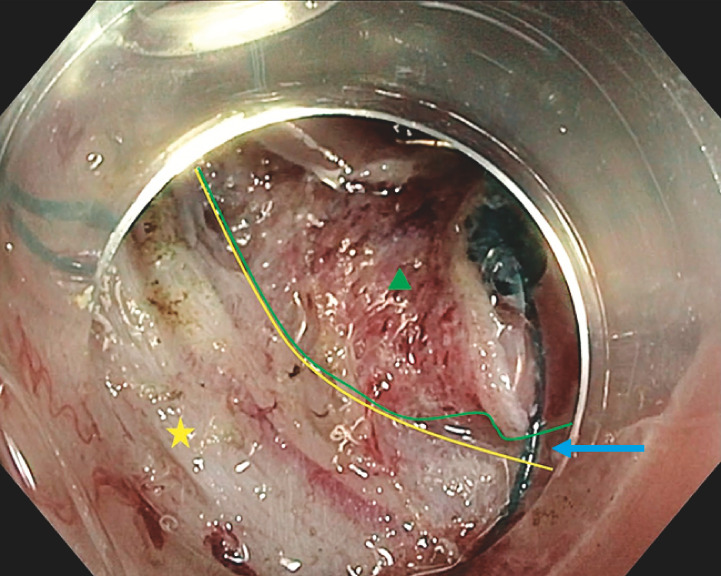
Endoscopic view of the diagnostic endoscopic submucosal dissection after tightening of the A-TRACT-2 device, allowing exposure on the very thin submucosal space (blue arrow) between the muscle layer (yellow star, bounded by the yellow line) and the mucosa (green triangle, bounded by the green line).

Diagnostic endoscopic submucosal dissection of the non-granular laterally spreading tumor with focal degeneration area.Video 1

The final histology was an adenocarcinoma with 1500-micron submucosal invasion without lymphovascular invasion or budding. In view of the low risk of lymph node recurrence, monitoring was proposed 5 . In conclusion, this device allowed multipolar adaptive traction for diagnostic ESD of a colonic lesion with focal invasive area, improving exposure even when the remaining submucosal space was very limited due to tumor submucosal invasion.

Endoscopy_UCTN_Code_TTT_1AQ_2AC

## Notes

Endoscopy E-Videos https://eref.thieme.de/e-videos
*E-Videos* is an open access online section of the journal *Endoscopy* , reporting on interesting cases and new techniques in gastroenterological endoscopy. All papers include a high-quality video and are published with a Creative Commons CC-BY license. Endoscopy E-Videos qualify for HINARI discounts and waivers and eligibility is automatically checked during the submission process. We grant 100% waivers to articles whose corresponding authors are based in Group A countries and 50% waivers to those who are based in Group B countries as classified by Research4Life (see: https://www.research4life.org/access/eligibility/ ). This section has its own submission website at https://mc.manuscriptcentral.com/e-videos .
